# The Overlooked Part of Flexible Assertive Community Treatment—A Retrospective Study on Factors Related to Discharge from FACT for Clients with a Psychotic Disorder  

**DOI:** 10.1007/s10597-023-01115-z

**Published:** 2023-04-22

**Authors:** Eva de Bruijn, Eline C. Jochems, André I. Wierdsma, Yolande Voskes

**Affiliations:** 1 GGZ Breburg, Tilburg, The Netherlands; 2 Tilburg School of Social and Behavioral Sciences, Tranzo Scientific Center for Care and Welfare, Tilburg University, Tilburg, The Netherlands; 3 GGzE, Eindhoven, The Netherlands; 4 Erasmus MC, Rotterdam, The Netherlands; 5 Department of Ethics, Law and Humanities, Amsterdam UMC, Amsterdam, The Netherlands

**Keywords:** FACT, Discharge, Recovery, Planned, Psychotic disorders

## Abstract

Flexible assertive community treatment (FACT) is a recovery-based treatment and its manual describes discharge criteria for clients who are recovered. Yet research on discharge is lacking. In this retrospective and observational study, between 2009 and 2019, we explored how sociodemographic, clinical, and treatment factors are associated with planned discharge or no discharge. We included 1734 clients with a psychotic disorder of which 38.5% were discharged after a mutual decision that FACT was no longer necessary. Logistic regression analysis was used to create a discharge profile which was more favorable for discharged clients. They were older at the start of FACT, had lower HoNOS scores, were diagnosed with another psychotic disorder, and had fewer contacts with non-FACT members. Discharge is a part of FACT and is more common than anticipated. While this study provides preliminary answers, further research is necessary to better understand discharge and its associated factors.

## Introduction

Recovery and rehabilitation are important objectives of Flexible Assertive Community Treatment (FACT) (Van Veldhuizen & Bahler, 2013) and with it, FACT offers professional treatment in line with recovery practices (Van Hoof et al., 2014). FACT meets the three criteria of recovery-based treatment: being flexible and responsive, community-based and aimed at reciprocity, and cohesion and continuity (van Hoof et al., 2014). If needed, multidisciplinary, community mental health teams provide high-intensity treatment in the clients’ environment. FACT offers a combination of treatment, care, and rehabilitation and recovery practices, in line with the treatment guidelines for schizophrenia in the Netherlands (Van Veldhuizen & Bahler, 2013). The continuity of care within FACT facilitates recovery and rehabilitation according to Van Veldhuizen and Bähler (2013). “FACT aims to support the clients in their recovery process. The goal is for clients to function optimally and to participate in society in a way that appeals to them and that is safe.” (Van Veldhuizen & Bähler, 2013, p.37). Ultimately, when clients meet their recovery goals, discharge from FACT is possible and will be discussed as part of the final stage of FACT treatment (LeFebvre et al., 2018). Thus, ‘planned discharge’ can be viewed as an outcome-oriented definition of recovery - client and healthcare professionals make a shared decision that FACT is no longer needed to sustain recovery (Bellack, 2006).

The road to recovery can be a bumpy one for clients with severe mental illness (SMI) who are served by FACT, but recovery, and thereby discharge, is possible (van Veldhuizen & Bähler, 2013). SMI is defined as a psychiatric disorder that severely limits social functioning, and for which a client needs coordinated treatment (Delespaul, & de consensusgroep EPA, 2013). The symptoms, limitations, and need for treatment are long-lasting and structural. Around 60% of SMI clients have a psychotic disorder (Delespaul, & de consensusgroep EPA, 2013; Van Vugt et al., 2018), and over 80% of the clients diagnosed with schizophrenia fall in the SMI definition.

Several studies to date have investigated the course of recovery in clients with SMI served by FACT, yet only a handful have looked into (planned) discharge from FACT as a recovery outcome. Kortrijk et al. (2019) found six subgroups of clients within FACT: four stable subgroups (‘low problem-severity level’ (27%), ‘low medium problem-severity level’ (45%), ‘high medium problem- severity level’ (20%) and ‘high problem-severity level’ (4%)), a subgroup which improved (2%) and a subgroup which deteriorated (2%). For almost all FACT clients their psychosocial problems were found stable for two years. This is in line with the SMI definition that their problems are long-lasting and structural (Delespaul, & de consensusgroep EPA, 2013). Long-term prospects are better: 20–30 years after treatment 68% of the most severe SMI clients had mild to no problems and 76% had a ‘moderate to full life’ (Harding et al., 1987). SMI clients recover at a slower pace than other clients. Arrested personal and social development and (self)stigma contribute to stagnated recovery more than the actual diagnosis (van Hoof et al., 2014). Discharge from FACT as a final phase of recovery was not always the norm (Van Vugt et al., 2018), but things change. Clients who ask to be discharged and fit the criteria (See box 1) for a minimum of 2 years can be discharged according to the FACT manual (Van Veldhuizen & Bahler, 2013). Nevertheless, discharge appears to be neglected in most FACT studies and is only an afterthought in a few studies. These studies found discharge rates ranging from 10% to 20% (Nugter et al., 2016; Van Vugt et al., 2018); with discharge rates doubling from 10% to 20% between 2009 and 2014, most of them after a shared decision that FACT was no longer necessary. Discharge without a referral (3%) was less customary (Van Vugt et al., 2018). Drukker et al. (2013) found that clients received on average three different FACT- periods with a mean duration of 2.9 years. A FACT episode ended when a client did not receive FACT treatment for more than 2 months.

The discharge criteria include important recovery aspects such as daytime activities, housing, and support systems; yet it seems that discharge itself – i.e., not needing specialist mental health treatment anymore – is not seen as a recovery outcome in most studies to date (Bellack, 2006; Leamy et al., 2011; Whitley et al., 2015).

Little is known about which clients get discharged and which factors relate to discharge from FACT. In the current study, we investigated differences between clients who have a planned discharge versus those who had no discharge (yet) from FACT. We explore whether and how socio-demographic, clinical, and treatment factors relate to discharge or no discharge from FACT. Furthermore, this study investigated how planned discharge or no discharge relates to some elements of FACT (VanVeldhuizen, 2013), namely multidisciplinary treatment, the intensity of treatment, and contacts that took place in the community.

Box 1 **Criteria to discharge patients from FACT**

Clients can be discharged if they wish to and meet the following criteria for at least two years:

- Fewer than 12 contacts per year, not focused on change
- No complex medication use
- An adequate support system, assessed by the team
- Work or daytime activities, assessed by the team
- Independent housing
- Organized financial situation

And if the client

- Accepts treatment if needed
- Is able to ask for help
- Has a primary care professional who is willing to monitor him or her

Discharge is also allowed for clients, who want to be discharged and do not meet the criteria; if there are no adverse effects for the client. (Van Veldhuizen, & Bähler, 2013, p.50)

## Methods

### Setting

Data came from ten FACT teams in the mid-south or the Netherlands (GGZ Breburg); after a reorganization at the beginning of 2016 eight teams remained. Each team was responsible for approximately 200 clients. Except for younger clients with a first psychosis, all clients with a psychotic disorder were assigned to a FACT team regardless of their SMI status and without complying with SMI criteria. Compared to other organizations these teams were more specialized in treating psychotic disorders and part of the regional Centre for Psychotic Disorders. Internal discharge criteria were in line with the discharge criteria in box 1. The criteria were stricter on the number of contacts (maximum of 6 contacts/year, not focused on change) and less strict on a period of stability (a minimum of 1 year). If clients used complex medication, the general practitioner (GP) wayed in on a potential discharge. When a GP did not want to prescribe the medication, discharge was not possible.

### Study Design and Procedure

**Fig. 1:**
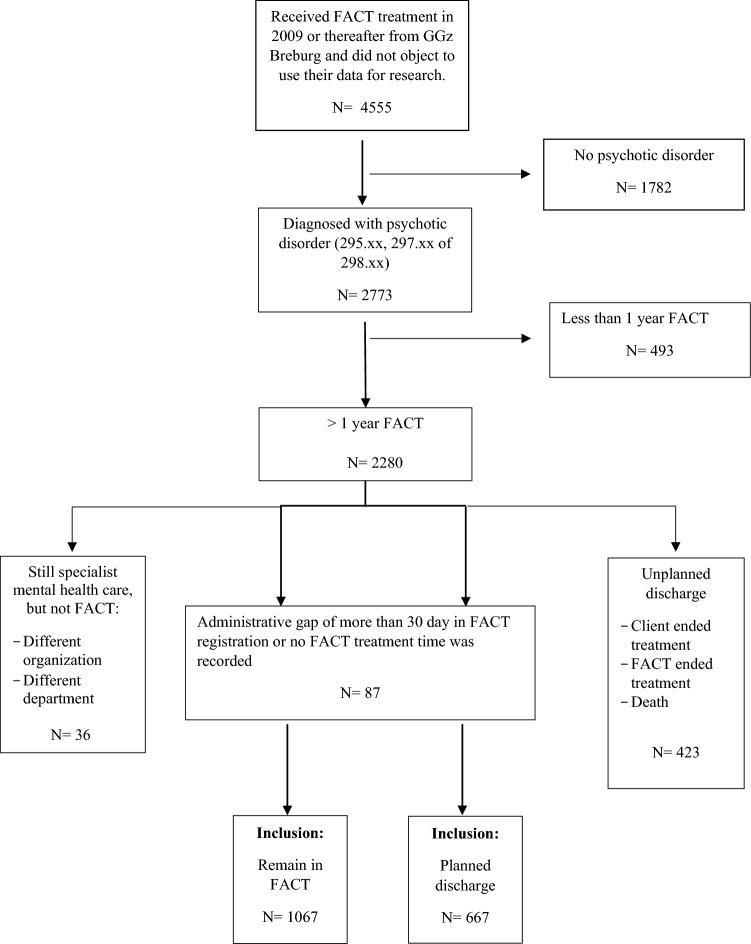
Flowchart inclusion clients in study

Using a retrospective, observational study design, we collected administrative data from the electronic files of clients with a psychotic disorder who received FACT treatment between 2009 and 2019. Client records met the following criteria to be included (see Fig. 1):

- the client did not object to using record data for scientific research,
- received a minimum of one year FACT treatment in 2009 or thereafter and did not have an administrative gap for more than 30 days,
- was diagnosed with a psychotic disorder in the first year of FACT (in DSM-IV-TR or DSM 5),
- was still in FACT care by September 2019 (‘no discharge’), or was previously discharged from FACT/specialist mental health care after a mutual decision made by the team and client that FACT was no longer necessary (‘planned discharge’). The reason for discharge (e.g. mutual decision or clients’ request) was registered by the involved practitioner.

### Measures

Socio-demographic, clinical, and factors related to treatment use were collected from the records. *Socio-demographic factors* included age at start of FACT, gender, and ethnicity. *Clinical factors* were: diagnosis, comorbidity, hospitalization in the first year of FACT, and Health of the Nation Outcome Scales (HoNOS, 0**-**48) total scores (Mulder et al., 2004) at start of FACT (first registered HoNOS in FACT episode).* Treatment use* in the first year of FACT was measured by several parameters, including the number of contacts provided by other teams in the organization and elements of FACT

The first FACT-element extracted from the medical records was the *percentage of contacts in the community*, defined as the number of contacts provided in the community as a percentage of total FACT-contacts (face to face, by telephone, and/or via e-health). The second element is *multidisciplinarity*, defined as the number of disciplines that the client had spent an average of 30 minutes with per contact (face to face, by telephone, and/or via e-health) during a year of treatment. This could range between 0 and 7 as there were seven disciplines marked: psychiatrist, psychologist, employment specialist, social worker, peer counselor, nurses (including case managers and social psychiatric nurses) and others. Time spent with a psychiatrist was included if the contact with a psychiatrist, face-to face or otherwise, had an average of 20 minutes per contact such that it could be considered actual consultation. The third parameter for treatment use was *intensity*, defined as the total number of treatment hours (face-to-face, by telephone, and/or via e-health) provided by members of the FACT team.

### Statistical Analysis

Descriptive statistics were used to compare the two groups (no discharge and planned discharge) regarding socio-demographic and clinical characteristics and treatment factors. Age, percentage of contact in the community, and contacts with other teams were categorized following clinical reasoning.

Logistic regression analysis was used to create a discharge profile in a stepwise procedure following Hosmer and Lemeshow (2000). Model selection was based on Wald-tests with alpha set at 0.25 and 0.05 for variable inclusion and elimination respectively. Model fit was evaluated based on Nagelkerke’s R-square, AUC-index, and the H&L-test. All analyses were conducted using IBM SPSS 26.

### Ethical Consideration

Prior to the start of the study, the study design was approved by the institutional review board of GGz Breburg (CWO2018-12). The collected data is administrative data that cannot be linked to a client by the researchers. Because of the large number of clients, and retrospective study design, it is not achievable to get permission from each client. Those whose objection is registered were not included. There are no known conflicts of interest and all authors certify responsibility.

## Results

### Sample Characteristics

The total sample of 1734 clients in this study consisted of 1128 (65.1%) male clients. Most clients (42.8%) were between 35 and 50 years of age at start of FACT. The majority was Dutch (70,5%), and had no comorbid psychiatric diagnosis (58%) according to the client record. Schizophrenia was the most common psychotic disorder (46.8%), followed by other psychotic disorders (39.6%) and schizoaffective disorder (13.6%). 1067 (61,5%) clients had no discharge (yet) and 677 (38,5%) clients had a planned discharge during the study time window. The overall median time, in FACT, was 4.63 (1–10) years, whereas the clients who were discharged had a median time of 3.33 (1–9.58) years, and the no discharge clients a median of 6.92 (1.08–10.00) years.

Discharged clients were more often non-Dutch, had lower HoNOS scores, were less often hospitalized, and were more likely to be diagnosed with another psychotic disorder and less likely to be diagnosed with schizophrenia than clients who remained (see Table 1). They used less care: fewer contacts with non-FACT members, fewer hours of care by FACT, and saw fewer team members than those who were still in FACT.

**Table 1:** Baseline sociodemographic, clinical, and care use variables according to discharge status

Characteristics	No discharge		Planned discharge	
	N	%	N	%
Age				
< 35 years	308	28.9	174	26.1
35–50 years	462	43.3	274	41.1
> 50 years	297	27.8	219	32.8
Male	698	65.4	430	64.5
Ethnicity				
Dutch	777	72.8	446	66.9
Non-Dutch	290	27.2	221	33.1
Diagnosis				
Schizophrenia	544	51.0	268	40.2
Schizoaffective disorder	151	14.2	85	12.7
Other psychotic disorders^a^	372	34.9	314	47.1
Comorbidity (Yes)	468	42.9	271	40.6
HoNOS score start FACT				
Score 0–5	213	20.0	196	29.4
Score 6–9	243	22.8	142	21.3
Score 10–13	187	17.5	100	15
Score > 13	405	38.0	162	24.3
Missing	19	1.8	67	10.0
Hospitalized in first year FACT (yes)	260	24.2	98	14.7
Number of contacts with non-FACT members				
0–12 contacts	42	3.9	101	15.1
13–52 contacts	411	38.5	344	51.6
> 53 contacts	614	57.5	222	33.3
% of contacts in the community^b^				
0%–20%	552	51.7	372	55.8
> 20%	515	48.3	295	44.2

	N	Median	LQ-HQ	N	Median	LQ-HQ
Intensity: hours of contact	1067	32.30	.60–1037.30	667	18.70	.30–829.50
Multidisciplinarity contact	1067	3	0–6	667	2	0–7

^a^Delusional disorder, brief psychotic disorder, psychotic disorder nos, schizophreniform disorder

^b^Face-to-face, telephone and e-health contacts

### Discharge Profile

The final model consisted of four variables to describe the discharge profile (see Table 2). Higher age at start FACT, being diagnosed with another psychotic disorder, low HoNOS scores, and few contacts with non-FACT members were positively related to the probability of a planned discharge. The clients who remained were younger when they entered FACT, were more often diagnosed with schizophrenia, had higher HoNOS scores, and had more specialized care contacts outside of FACT. None of the three elements of FACT treatment (namely percentage of contacts in the community, multidisciplinarity, and treatment intensity) were sustained in the final model. The interaction effects (between diagnosis and age and between diagnosis and first HoNOS score) that were expected to be of clinical relevance with regards to discharge status, also did not sustain in the final parsimonious model.

**Table 2:** Factors related to discharge (parsimonious model)

		95% CI for Exp(B)		
	Beta (SE)	Lower	Exp(B)	Upper
Constant	1.712 (.250)		5.539	
Age				
< 35 years	− .307 (.143)*	.556	.736	.974
35–50 years	− .285 (.129)*	.585	.752	.967
> 50 years	Ref. cat			
Psychotic disorder				
Schizophrenia	− .698 (.118)**	.394	.497	.627
Schizo-affective	− .578 (.170)**	.402	.561	.783
Other psychotic disorders	Ref. cat			
HoNOS Start				
Score 0–5	Ref. cat			
Score 6–9	− .383 (.151)*	.507	.682	.918
Score 10–13	− .461 (.166)**	.455	.631	.873
Score 13 +	− .773 (.144)**	.348	.461	.612
Contacts with other teams				
0–12	Ref. cat			
13–52	− .990 (.215)**	.244	.372	.566
> 53	− 1.681 (.217)	.122	.186	.284

R2= .127 (Nagelkerke); AUC =.680 CI .653-.706, p <.000; Hosmer & Lemeshow Goodness of Fit = 2.777, p= .948

*P<.05, **P<.01

## Discussion

Discharge from FACT seemed to be an overlooked outcome of effective treatment for clients with psychotic disorders (van Vugt, 2015). In this study we found that more than a third of clients were discharged, most of them within four years. Planned discharged clients had a more favorable profile in the first year of FACT than those who did not. Lower HoNOS score and less contact with non-FACT members point to ‘milder’ symptoms. This is in line with findings that the severity of symptoms in the first two years is a predictor of long-term outcome (Harrison et al., 2001). Furthermore, discharged clients had more often a non-schizophrenic psychotic disorder, which is linked to better outcomes than those with a schizophrenic disorder (Harrow et al., 1997; Harrison et al., 2001). Finally, clients who entered FACT at an older age were discharged more often. Both positive and negative effects are reported when it comes to the age of onset of psychotic symptoms and outcome (Immonen et al., 2017). The positive effect of older age at start FACT on discharge, in this study, may be due to better premorbid functioning, milder symptoms, a non-schizophrenic disorder, better resilience, and/or a better social network.

The three elements of FACT, namely intensity, multidisciplinarity, and contacts in the community, along with other treatment factors (contacts with non-FACT members excluded) were not found to be part of the discharge profile. It thus appears that the type of treatment in the first year of FACT does not relate to discharge, despite the assumption that more intense, multidisciplinary community mental health care would be beneficial for recovery (and thus discharge status). Three explanations could be considered. First, in this study, treatment use was operationalized as use in the first year of FACT, with a median of more than four years (over the total sample). For clients whose discharge takes place after several years of treatment, this ‘baseline’ use of treatment is no longer related to discharge status. This is in line with the findings that 80% of FACT clients alternate over two years between periods of intensive, shared caseload treatment and individual case management (Van Veldhuizen, 2007), and in line with the research of Kortrijk et al. (2019) that patterns of care use are better predictors of the type of discharge. Second, our findings are in line with the flexibility of FACT where teams match the level of treatment to the needs of the clients, such that clients with milder symptoms may need less intensive, multidisciplinary treatment in the community. All clients with a psychotic disorder are treated in FACT, even though not all clients need the elements of FACT to recover and be discharged. Finally, there may not be a direct effect of the elements on discharge. Yet it is clinically plausible that the elements of FACT interact with clinical factors such as the severity of symptoms, level of support from the client’s network, and/or premorbid functioning to determine recovery and (timing of) discharge. This could be investigated in future studies, to explore which elements of FACT contribute (most) to recovery in clients with psychotic disorders.

Further, psychotic disorder diagnoses may vary across 10 years, due to new insights, as it is known that more clients with other psychotic disorders get rediagnosed with schizophrenia and schizoaffective disorders over time (Bromet et al., 2011). Taking this into account, it is possible that in both discharge groups clients were rediagnosed with schizophrenia during the total study time and that this creates a potential bias in this study. Changes in diagnoses over time could be linked to differences in discharge, which is worth investigating in the future.

Discharge rates may vary over time. Van Vugt et al. (2018) found that discharge rates increased at the same time that the organization of the Dutch mental health system was altered, such that basic mental health care opened up for stable, low-severity FACT clients. This form of healthcare consists of low frequency contacts provided by a mental healthcare professional in the general practitioner’s office or generalized mental health care organization. Basic mental health care can be a suitable alternative for some FACT clients and keeps specialist mental health care by FACT available for the less stable clients. Further research is recommended to study the effect of basic mental health care on discharge.

This is the first study on discharge from FACT, and although it provides first insights into discharge, it raises many more questions about discharge and related factors. For example, how do patterns of treatment use relate to discharge, and are there differences in discharge number/patterns between FACT- teams? Also, what happens to clients who need a lot of care in the first year of FACT; are they more likely to have a schizophrenic disorder, and to fall into the ‘stable high severity level’ (Kortrijk et al., 2019), and (when) do they discharge with successful recovery outcomes? Furthermore, in line with Nordén and Norlander (2014), further research into FACT elements and their effect on recovery and discharge is indicated.

There are some limitations in this study. The dataset had not the necessary extensiveness to answer the questions arising from this study. Data on readmission was lacking. It is unclear how many FACT- episodes each client had and how many were readmitted after a successful discharge. For future research, it is advised to include this in the study. The observational and retrospective nature of this study implies that the data was not recorded for research purposes and therefore can lack (inter-rater) reliability or validity. For instance, we found lower numbers of comorbidity than in other studies (Kortrijk et al., 2019). This could be due, for example, to administrative complexity or that the first diagnosis could be a working hypothesis rather than a definitive diagnosis. In addition, using only administrative data has its limitations. For example, not having access to all discharge criteria, as mentioned in the FACT manual (see box 1), including information such as an adequate support system, housing, and employment were not available, whereas these are found to be linked to recovery (Leendertse et al., 2021). While this study is unable, due to the design, to directly asses that clients meet the discharge criteria, and thereby, the criteria for (a successful) recovery, they were discharged according to the internal discharge criteria. Which implies that there was multidimensional recovery.

The percentage of contacts in the community was lower than expected due to the collection of all contacts with the client. This is in line with blended treatment practices. Contacts by telephone and e-health, which can be common, were not defined as contact in the community. Nevertheless, they were used to calculate the percentage of contacts in the community, and by doing so lowering the percentage of face-to-face contacts in the community. Furthermore, HoNOS scores were lower than expected, which could imply a healthier sample than in other studies and clinical settings. This could be a result, among other things, of the inclusion criteria used in the studied FACT-teams. All clients with a psychotic disorder were included, regardless of the severity of their symptoms. Finally, this study explored discharge patterns from FACT in a Dutch health care setting and future research will show if the factors that were found to be relevant to discharge in this study will also apply in other countries with different health care organizations. The purpose of this study was to identify which socio-demographic, clinical, and treatment factors relate to discharge. Based on the analysis conducted, a discharge profile was created with four socio-demographic, clinical, and treatment factors in the first year of FACT. The three elements of FACT in the first year had no part in the discharge profile. Further research into the factors and treatment use patterns could be useful to better understand who the discharged clients are and how FACT treatment could be organized to serve the individual needs better to promote recovery and planned discharge.

## Data Availability

Due to the nature of this research, participants of this study did not agree for their data to be shared publicly, so supporting data is not available.
